# Physical Activity and Perceived Insecurity from Crime in Adults: A Population-Based Study

**DOI:** 10.1371/journal.pone.0108136

**Published:** 2014-09-24

**Authors:** Márcio de Almeida Mendes, Inácio Crochemore Mohnsam da Silva, Pedro Curi Hallal, Elaine Tomasi

**Affiliations:** 1 Graduate Program in Epidemiology, Federal University of Pelotas, Rio Grande do Sul, Brazil; 2 Graduate Program in Epidemiology, Federal University of Pelotas, Rio Grande do Sul, Brazil; Old Dominion University, United States of America

## Abstract

**Background:**

The present study aimed to describe the perception of safety from crime in the neighborhood and to evaluate its association with leisure-time and transport-related physical activity in adults.

**Methods:**

A cross-sectional population based study was conducted in the urban area of Pelotas, Brazil, in 2012. Perceived insecurity from crime in the neighborhood was measured using the *Neighborhood Environment Walkability Scale* (NEWS) and the *City Stress Inventory* (CSI). Physical activity was measured using an adapted version of the leisure and transportation sections of the long version of the International Physical Activity Questionnaire.

**Results:**

Overall, 52.3% (95%CI49.0; 55.6) of the participants reported perceived exposure to an unsafe neighborhood. Subjects who practiced 150 or more minutes per week of physical activity during leisure-time and transportation were 10.5% (95%CI9.0; 12.0) and 51.7% (95%CI 48.7; 54.7), respectively. There were no significant associations between physical activity (leisure-time or transport-related) and perceived insecurity from crime, neither in unadjusted nor in adjusted analyses.

**Conclusion:**

There was no evidence that the perception of safety from crime is associated to higher physical activity levels among Brazilian adults.

## Introduction

Over 5 million annual deaths worldwide are attributable to physical inactivity [1], a risk factor that affects one third of the world's adult population [2]. For public health, understanding the correlates and determinants of this behavior is therefore essential [3]. Until two decades ago, there were few studies linking environmental characteristics with physical activity practice [3]. Most correlates investigated at that time were based on theoretical models emphasizing psychological and social influences on behavior, not considering aspects that go beyond the individual level [4].

More recently, ecological models have gained widespread attention in the field, emphasizing that one's behavior is influenced by factors ranging from the individual to the macro-societal levels [5]. Consequently, several studies have explored the influence of environmental factors on the development or maintenance of healthy behavior, characterizing the individuals' interaction with the environment as an important tool to understand complex behaviors such as physical inactivity [3], [6], [7].

In this context, topics like population growth, insecurity related to traffic, air pollution and access to parks and other facilities have been identified as important correlates of physical activity [8]. Perceived insecurity from crime is another characteristic that has been studied as a possible correlate of physical activity, although the findings on this association remain inconsistent [9]. Furthermore, leisure-time and transport-related physical activity have different correlates [10] and studies reporting findings specific to each domain are desirable. Additionally, few studies from low and middle-income countries are available, exactly the parts of the world in which insecurity from crime is more of a major public health threat [11]. For example, the average homicide rate worldwide was estimated at 7.9 per 100,000 people, whereas in Brazil the equivalent rate was 29.6 per 100,000 people [12].

The aim of the present study was to describe the perception of insecurity from crime in the neighborhood and to evaluate its association with leisure-time and transport-related physical activity in adults living in Pelotas, Brazil.

## Methods

The Research Ethics Committee of the Federal University of Pelotas Medical School approved the study protocol (n°. 17/11) and written informed consent was obtained from each participant. This study is part of a multipurpose health survey, further details about its methodology are available in a previous publication [13]. It was a cross-sectional population based study carried out in the urban area of Pelotas, Brazil in 2012. The city is located in the south of Rio Grande do Sul state, with a population of about 300,000 inhabitants in the urban zone [14].

The sampling process was conducted in multiple stages. The primary sampling units were 495 census tracts defined by the Brazilian Institute of Geography and Statistics (IBGE). Each census tract contains approximately 300 households. In total, 130 census tracts were randomly sampled and around 13 households per tract were systematically selected. All individuals aged 20 years or older, living in the sampled households, were eligible for the study, with the exception of those with serious physical or mental impairment that impeded them to answer to the questionnaire. At least three attempts with each sampled individual were done before considering him/her as a refusal; no replacement was done. The final sample is representative of the city's population; the sample distribution in terms of sex, age and income is virtually identical to that of the city's population based on census data [14].

The outcome variables leisure-time and transport-related physical activity were assessed through an adapted version of the leisure and transportation sections of the long version of the International Physical Activity Questionnaire (IPAQ) [15]. Transport-related physical activity was measured using questions referring to walking or bicycle riding. Leisure-time physical activity was measured using questions related to frequency and duration of walking, running and bicycle riding.

Individuals who reported 150 minutes or more per week of leisure-time physical activity were considered active in this domain [16]. In terms of transport-related physical activity, those reporting to use an active mode of transportation at least once per week were considered active. In both domains, only activities with a minimum duration of 10 minutes were considered. For those subjects who reported to practice leisure-time physical activities, we inquired how distant (if near or far) from their households these activities were practiced.

The questions on the perception of insecurity from crime were based on the “*safety from crime*” domain from the *Neighborhood Environment Walkability Scale* (NEWS), an instrument that has been validated in many countries, including Brazil [17]. These questions provide information about the individuals' perception regarding criminality and insecurity for walking in the neighborhood. Questions from the *City Stress Inventory* (CSI) [18], a scale about disorders and exposure to violence in the neighborhood, were used as well. “Neighborhood” or sentences like “near your house” were defined as places for which the participants could walk in less than 15 minutes. The questions about insecurity were: *1) “Near your house, how many times do mild crimes, such as wall graffiti, public telephones destruction, or small thefts like bicycles, gas cylinders or lamps thefts, happen?; 2) Near your house, do serious crimes like armed robbery, burglary, and assaults happen?; 3) Near your house, does buying and sale of drugs happen?; 4) During the day, near your house, do you feel safe to walk, ride a bicycle or do sports? 5) During the night, near your house, do you feel safe to walk, ride a bicycle or do sports?”* Each of the five questions presented four categories of response: *a) never; b) sometimes or few times; c) many times or almost always; d) I do not know*. Perceived insecurity from crime was defined as when individuals answered the option *many times or almost always* in, at least, one of the three first questions, or the option *never* in, at least, one of the two last ones.

Data were collected through face-to-face interviews, conducted by trained personnel. Interviewers were trained for 40 hours prior to data collection. Pilot studies were conducted in order to evaluate the understanding of the questionnaire by the respondents, as well as took place as the last stage of the training of the interviewers. Quality control was performed by revisiting a random 10% of the sampled individuals. For this group, a summary version of the research instrument was applied, for an evaluation of agreement using the *kappa* statistic. The questions about insecurity from crime presented a kappa value of 0.84.

Confounding variables included in the regression models were: sex (male, female), age (in decades), self-report skin color (white, non-white), schooling level (number of years with approval and categorized as 0–4, 5–8, 9–12, ≥13), household *per capita* income (divided into quintiles) and place of residence in the city of Pelotas (center or periphery).

Data analysis was carried out in *Stata12.1*, using the *svy* group of commands in order to account for the complex survey design. In the unadjusted analysis, chi-square tests for heterogeneity and linear trend were conducted. Multivariable Poisson regressions were run for both outcomes (leisure-time and transport-related activity). We also tested the interactions between perceived insecurity from crime and (a) sex; (b) age; (c) family income. For all the analyses, a significance value of 5% was adopted.

## Results

Within the 1,722 selected households, 3,326 adults were considered eligible for the study, and of them, 2,874 answered to the questionnaire. The response rate was 86.4% (452 losses and refusals).Most participants were female (58.8%), aged between 20 and 49 years old (60.2%), had white skin color (82.6%), had more than eight years of schooling (54.5%), and were residents from the periphery of the city (80.7%). Regarding income, the individuals from the first quintile (poorest) earned up to R$ 344.10 and the ones from the last quintile (wealthiest) earned from R$ 1,440.28 to R$ 20,000.00 (One Brazilian Real equivalent to 2.29 American Dollars as of August, 2013). There was a strong positive association between schooling and income.

From the participants, 52.3% (95%CI 49.0; 55.6) were classified as exposed to insecurity from crime in their neighborhoods, while the prevalence of physical activity was 10.5% (95%CI 9.0; 12.0) during leisure time and 51.7% (95%CI 48.7; 54.7) during transportation (Table 1).

**Table 1:** Sample description and the prevalence of physical activity according to demographic, socioeconomic and environmental variables.

Variables	N	%	Leisure-time physical activity	*p*	Transport-related physical activity	*p*
			Prevalence (_95%_CI)		Prevalence (_95%_CI)	
**Gender**				0.001*		0.053*
Male	1,184	41.2	13.0 (11.0 – 15.1)		49.6 (46.0 – 53.1)	
Female	1,690	58.8	8.7 (7.1 – 10.4)		53.2 (49.7 – 56.7)	
**Skin color**				0.144*		0.239*
White	2,375	82.6	10.9 (9.3 – 12.5)		51.2 (48.1 – 54.2)	
Non-white	499	17.4	8.6 (6.0 – 11.3)		54.3 (48.8 – 59.8)	
**Age (years)**				0.176*		0.001**
20–29	607	21.2	10.7 (7.7 – 13.7)		58.6 (53.7 – 63.4)	
30–39	535	18.6	7.5 (5.2 – 9.8)		49.0 (44.3 – 53.7)	
40–49	586	20.4	10.9 (8.1 – 13.7)		52.0 (47.0 – 56.9)	
50–59	507	17.6	12.5 (9.4 – 15.6)		52.8 (47.7 – 57.8)	
60 or more	639	22.2	10.8 (7.8 – 13.8)		46.4 (41.7 – 51.1)	
**Schooling (years of study)**				0.001**		0.494*
0 – 4	503	17.6	5.2 (3.0 – 7.4)		50.0 (45.3 – 54.7)	
5 – 8	801	27.9	9.1 (6.7 – 11.6)		50.0 (45.6 – 54.5)	
9 – 12	897	31.2	11.3 (9.0 – 13.5)		53.0 (49.1 – 56.9)	
13 or more	670	23.3	15.1 (11.9 – 18.3)		53.4 (48.1 – 58.6)	
***Per capita*** ** income (quintiles)** &				0.001*		0.001**
1° - Until R$ 344,10	564	20.3	10.0 (6.9 – 13.0)		57.6 (51.8 – 63.5)	
2° - R$ 344,11 to 580,50	551	19.8	7.1 (4.4 – 9.7)		51.3 (46.2 – 56.4)	
3° - R$ 580,51 to 807,33	553	19.9	11.1 (8.2 – 14.0)		53.0 (48.2 – 57.7)	
4° - R$ 807,34 to 1440,27	552	19.8	8.5 (6.3 – 10.8)		51.0 (45.2 – 56.8)	
5° - R$ 1.440,28 to 20.000,00	562	20.2	15.5 (11.8 – 19.2)		46.3 (40.8 – 51.9)	
Continuation						
**City areas of Pelotas**				0.002*		0.001*
Center	554	19.3	15.0 (11.5 – 18.5)		60.8 (55.0 – 66.5)	
Periphery	2,320	80.7	9.4 (7.8 – 11.0)		49.5 (46.2 – 52.9)	
**Total**	2,874	100	10.5 (9.0 – 12.0)		51.7 (48.7 – 54.7)	

Pelotas/RS-Brazil, 2012.

& One Brazilian Real equivalent to 2.29 American Dollars (exchange rate used: August 2, 2013).

*Chi-square test of heterogeneity.

** Chi-square test for linear trend.

There were associations between leisure-time physical activity and the variables sex (*p* = 0.001), schooling (*p* = 0.001), household *per capita* income (*p* = 0.001) and place of residence (*p* = 0.002). The most active groups were men [13.0% (95%CI 11.0; 15.1)], the wealthiest income quintile [15.5% (95%CI 11.8; 19.2)] and residents in the city center [15.5% (95%CI 11.5; 18.5)]. Leisure-time physical activity also increased with the number of years of schooling (Table 1). In relation to transportation, associations were found with age (*p* = 0.001), income (*p* = 0.001), and place of residence (p = 0.001), being more active the individuals living in the city center [60.8% (95%CI 55.0; 66.5)]. Additionally, transport-related physical activity decreased with age and income (Table 1).

Perception of frequent (described as “many times” or “always”) drug trade in the neighborhood was mentioned by a quarter of the participants. Perception of frequently occurrence of mild and serious crimes was reported by 13% and 10% of the sample, respectively. Around a third reported perceived insecurity many times or always while practicing physical activity at night. In the bivariate analysis, none of the five variables about perception of insecurity from crime presented a significant association with physical activity (leisure-time or transportation) (Table 2).

**Table 2:** Sample description and the prevalence of physical activity according to the variables related to perceived safety from crime.

Variables	N	%	Leisure-time physical activity	*p*	Transport-related physical activity	*p*
			Prevalence (_95%_CI)		Prevalence (_95%_CI)	
**Mild crimes**				0.118*		0.622*
Never	1,168	40.7	9.0 (7.0 – 11.0)		50.9 (47.1 – 54.6)	
Sometimes or few times	1,327	46.3	11.6 (9.7 – 13.6)		52.7 (49.1 – 56.3)	
Many times or almost always	372	13.0	11.1 (7.6 – 14.7)		50.9 (45.3 – 56.6)	
**Serious crimes**				0.313*		0.567*
Never	1,310	45.6	11.3 (9.2 – 13.4)		52.6 (49.0 – 56.2)	
Sometimes or few times	1,271	44.3	10.2 (8.2 – 12.2)		50.6 (47.0 – 54.2)	
Many times or almost always	291	10.1	8.3 (4.9 – 11.6)		52.8 (46.0 – 59.5)	
**Buying and sale of drugs**				0.401*		0.663*
Never	1,703	59.3	10.8 (8.9 – 12.7)		51.0 (47.5 – 54.4)	
Sometimes or few times	449	15.7	11.6 (8.6 – 14.6)		52.6 (47.2 – 57.9)	
Many times or almost always	717	25.0	9.1 (6.5 – 11.7)		53.0 (48.2 – 57.8)	
**Safety for physical activity during the day**				0.731*		0.611*
Never	182	6.4	8.8 (4.6 – 13.1)		48.4 (41.2 – 55.5)	
Sometimes or few times	412	14.4	10.4 (7.6 – 13.3)		51.5 (45.5 – 57.4)	
Many times or almost always	2,269	79.2	10.6 (9.0 – 12.2)		52.1 (49.0 – 55.3)	
**Safety for physical activity during the night**				0.521*		0.193*
Never	972	34.2	9.7 (7.4 – 11.9)		54.6 (50.6 – 58.5)	
Sometimes or few times	813	28.6	11.2 (9.1 – 13.3)		50.4 (45.6 – 55.2)	
Many times or almost always	1,056	37.2	10.7 (8.7 – 12.8)		50.8 (46.9 – 54.7)	
Continuation						
**Insecurity from crimes#**				0.173*		0.207*
No	1,347	47.7	11.3 (9.4 – 13.2)		50.5 (46.7 – 54.4)	
Yes	1,477	52.3	9.6 (7.8 – 11.5)		53.3 (49.7 – 56.9)	

Pelotas/RS-Brazil, 2012.

*Chi-square test of heterogeneity.

#Score combined with the five previous variables.

Perceived insecurity from crime presented significant associations with sex (*p* = 0.001), schooling (*p* = 0.001) and income (*p* = 0.004). Women reported 33% higher perceived insecurity from crime than men [Prevalence Ratio (PR) = 1.33 (95%CI1.24; 1.43)]. Perceived security from crime increased with years of schooling and income (Table 3). In the adjusted analysis, no associations between perceived insecurity from crime and leisure-time (*p* = 0.716) or transport-related (*p* = 0.508) physical activity were found (Table 4).

**Table 3:** Prevalence of insecurity from crimes according to demographic, socioeconomic and environmental variables.

Variable	Insecurity		(_95%_CI)	*p*
	N	%		
**Gender**				0.001*
Male	510	43.7	(40.0 – 47.5)	
Female	967	58.3	(54.7 – 61.9)	
**Skin color**				0.145*
White	1,203	51.6	(48.3 – 54.9)	
Non-white	274	55.7	(49.6 – 61.5)	
**Age (years)**				0.147*
20–29	298	49.8	(44.7 – 54.8)	
30–39	299	56.6	(51.5 – 61.8)	
40–49	302	52.8	(47.7 – 57.9)	
50–59	248	49.2	(43.7 – 54.7)	
60 or more	330	53.1	(48.1 – 58.0)	
**Schooling (years of study)**				0.001**
0 – 4	274	56.0	(50.6 – 61.2)	
5 – 8	429	54.4	(49.7 – 59.0)	
9 – 12	469	53.5	(49.2 – 57.8)	
13 or more	304	45.7	(40.7 – 50.6)	
***Per capita*** ** income (in quintiles)**				0.004**
1° - Until R$ 344.10	319	57.4	(51.7 – 62.9)	
2° - R$ 344.11 to 580.50	285	52.5	(46.3 – 58.7)	
3° - R$ 580.51 to 807.33	296	54.1	(47.9 – 60.3)	
4° - R$ 807.34 to 1440.27	264	49.1	(43.5 – 54.7)	
5° - R$ 1,440.28 to 20,000.00	272	49.3	(43.3 – 55.2)	
Continuation				
**City areas of Pelotas**				0.002*
Center	328	59,9	(55.5 – 64.2)	
Periphery	1,149	50,5	(46.6 – 54.3)	
**Total**	1,477	52.3	(49.0 – 55.6)	

Pelotas/RS-Brazil, 2012.

*Chi-square test of heterogeneity.

**Chi-square test for linear trend.

**Table 4:** Association between leisure-time and transport-related physical activity and insecurity from crime.

Leisure-time physical activity				
Variables	Crude analysis		Adjusted analysis†	
	Prevalence ratio (_95%_CI)	*p*	Prevalence ratio (_95%_CI)	*p*
**Insecurity from crime**				
No	1.00	0.173*	1.00	0.716&
Yes	0.85 (0.68 – 1.07)		0.96 (0.74 – 1.23)	

Transport-related physical activity				
Insecurity from crime		**0.207** *		**0.508** &
No	1.00		1.00	
Yes	1.05 (0.97 – 1.15)		1.03 (0.95 – 1.11)	

Pelotas/RS-Brazil, 2012.

*Chi-square test of heterogeneity.

& Wald test of heterogeneity.

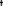
Adjustment for sex, skin color, age, schooling, income and place of residence.

Even when the analysis was restricted to the individuals that reported leisure-time activity practice near their residences, no associations were found, neither in crude (*p* = 0.954) nor in adjusted analysis (*p* = 0.823) (Table 5). Finally, no interactions between insecurity from crime and (a) sex; (b) age; (c) income at determining physical activity were detected.

**Table 5:** Association between leisure-time physical activity and insecurity from crime, restricted to individuals who reported practicing these activities close their neighborhoods.

	Crude analysis		Adjusted analysis†	
Variable	Prevalence ratio (_95%_CI)	*p*	Prevalence ratio (_95%_CI)	*p*
**Insecurity from crimes**				
No	1.00	0.954*	1.00	0.823&
Yes	0.99 (0.81 – 1.23)		1.03 (0.82 – 1.28)	

Pelotas/RS-Brazil, 2012.

*Chi-square test of heterogeneity.

& Wald test of heterogeneity.

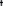
Adjustment for sex, skin color, age, schooling, income and place of residence.

## Discussion

Leisure-time physical activity decreased and transport-related physical activity increased, relative to previous studies in the same city [19], [20]. Nearly half of the participants (52.3%) reported insecurity in their neighborhoods in the current study. This prevalence might be considered high, but comparisons with other Brazilian cities are difficult because different instruments and operational definitions have been used in previous studies. The *International Crime Victims Surveys* (ICVS) measured perception of crimes such as vandalism, burglary, assaults and robberies. Using the ICVS, perceived insecurity between 2002 and 2005 in São Paulo and Rio de Janeiro were estimated at 20% and 15%, respectively [21].

One of the few sources of descriptive data about perceived security from crime, *Statistics Canada*, in the *General Social Survey* (GSS) 2009, showed that women, elderly individuals and those with low income had higher levels of insecurity. In this study, women and low-income individuals reported a higher perceived insecurity and an association with age groups was not found [22]. We were unable to use official statistics from the city, because they are not available. However, we understand that one's perception of insecurity is at least as important as official data at influencing behavior.

Previous studies investigated perceived safety from crime using questions such as “How safe do you feel walking in your neighborhood at night?” [23], or “Do you feel safe returning to your home when it is dark?” [24]. The phrasing of these questions implies threat, but do not define the source (e.g. crime, stray dogs, traffic). There is a risk that the question will be interpreted differently, making comparative analysis difficult and results unespecific [9]. For these reasons, we chose to use an instrument that clearly defined the source of the perceived threat as well as frequency of occurrence. Future studies could also consider collecting data about the length of time that the individual has lived in the neighborhood, since it might affect the perceived insecurity. Another possible explanation for our findings is the individual history of suffering a crime. A person with such history might have different perceptions about violence.

In the present study, associations between physical activity (leisure-time and transport-related) and perceived insecurity from crime were not observed in any of the analyses carried out. Instruments that specify the sources of insecurity, several operational definitions of the outcomes and exposure, as well as different types of interactions tested, all seem to strengthen the evidence that there is no association between physical activity and insecurity perception.

Florindo et al. conducted a population based study in the Brazilian south-eastern region. Physical activity was measured using the long version of IPAQ (leisure-time and transportation) and insecurity was measured using *NEWS*. Perceived security from crime was positively associated with transport-related physical activity [25]. The same conclusion was drawn in a study by Parra et al. conducted in Curitiba (PR), Brazil [26]. However, Amorim et al. did not find an association between perceived security from crime and transport-related physical activity in Pelotas (RS), Brazil, but found that leisure-time physical activity was positively associated with perceived security from crime [18]. With similar instruments to those used by Amorim et al. [19], Florindo et al. [25] and Parra et al. [26], but in a different region of Brazil, Hallal et al. [27]did not find associations between leisure-time and transport-related physical activity and perceived insecurity from crime.

The effect of the perceived insecurity on physical activity is complex and is likely affected by multiple levels of influence. Considering that the choice of a transport mode is dependent of factors other than individual preference only, suggests that safety concerns may be less important. Therefore, it is comprehensible that perceived insecurity from crime might not be a determinant of transport-related physical activity. Additionally, in terms of leisure-time physical activity, possibly the pleasure and habit of physical activity override any self-imposed limitation due to perception of insecurity from crime.

Foster et al. suggested that the lack of association between perceived insecurity and leisure-time physical activity could be explained by activities being carried out far away from the neighborhood. Thus, negative perceptions about the place of residence might not influence physical activity levels [9]. To test this hypothesis, an additional analysis was carried out excluding individuals reporting to carry out leisure-time physical activities far away from where they live. No difference in results was found.

Theoretically, perceived insecurity from crime has a higher influence on physical activity among women and elderly people [9]. However, associations were not found when interactions analyses by sex, age and income were tested. Analysis carried out separately for leisure-time and transport-related physical activity might be a possible explanation of these differences comparing with other studies.

## Conclusion

High levels of insecurity from crime were found in all population groups. These findings highlight the importance of strategies that improve the interaction between individuals and their living environments, to ensure wellbeing and a better quality of life of the population. Perceived insecurity from crime was not associated with physical activity in the present analysis. An important point to be highlighted is the publication of negative results such as those shown in this manuscript. Since papers with negative results can minimize publication bias. Future studies using prospective approaches, assessing specific sources of insecurity and evaluating interactions separately for each domain of physical activity (leisure-time and transportation) in the population could provide further evidence about this association.

## References

[1] Lee IM, Shiroma EJ, Lobelo F, Puska P, Blair SN, Katzmarzyk PT (2012) Effect of physical inactivity on major non-communicable diseases worldwide: an analysis of burden of disease and life expectancy. The Lancet380(9838): 219–29. 10.1016/S0140-6736(12)61031-9 PMC3645500 22818936

[2] Hallal PC, Andersen LB, Bull FC, Guthold R, Haskell W, et al (2012) Global physical activity levels: surveillance progress, pitfalls, and prospects. The Lancet 380(9838): 247–57. 10.1016/S0140-6736(12)60646-1 22818937

[3] Bauman AE, Reis RS, Sallis JF, Wells JC, Loos RJF, et al (2012) Correlates of physical activity: why are some people physically active and others not? The Lancet 380(9838): 258–71. 10.1016/S0140-6736(12)60735-1 22818938

[4] Sallis JF (2009) Measuring Physical Activity Environments: A Brief History. Am J Prev Med36(4): 86–92. 10.1016/j.amepre.2009.01.002 PMC2921817 19285214

[5] Humpel N, Owen N, Leslie E (2002) Environmental factors associated with adults' participation in physical activity: A review. Am J Prev Med 22(3): 188–99. 11897464 10.1016/s0749-3797(01)00426-3

[6] Hill JO, Peters JC (1998) Environmental Contributions to the Obesity Epidemic. Science 280(5368): 1371–4. 9603719 10.1126/science.280.5368.1371

[7] Wilson DK, Kirtland KA, Ainsworth BE, Addy CL (2004) Socioeconomic status and perceptions of access and safety for physical activity. Ann Behav Med 28(1): 20–8. 15249256 10.1207/s15324796abm2801_4

[8] World Health Organization. Global Strategy on Diet, Physical Activity and Health. Physical Inactivity: A Global Public Health Problem. [cited 2012 October 22]; Available from: http://www.who.int/dietphysicalactivity/factsheet_inactivity/en/.

[9] Foster S, Giles-Corti B (2008) The built environment, neighborhood crime and constrained physical activity: An exploration of inconsistent findings. Prev Med 47(3): 241–51. 18499242 10.1016/j.ypmed.2008.03.017

[10] HallalPCReisRSCerinESpecial Issue on Physical Activity and Safety from Crime. Journal of Environmental and Public Health; 2011 [October 20, 2012]; Available from downloads.hindawi.com/journals/jeph/si/pasfc.pdf.

[11] Reichenheim ME, de Souza ER, Moraes CL, de Mello Jorge MHP, da Silva CMFP, et al (2011) Violence and injuries in Brazil: the effect, progress made, and challenges ahead. The Lancet 377(9781): 1962–75. 10.1016/S0140-6736(11)60053-6 21561649

[12] Murray J, Cerqueira DR, Kahn T (2013) Crime and violence in Brazil: Systematic review of time trends, prevalence rates and risk factors. Aggress Violent Behav 18(5): 471–483. 24027422 10.1016/j.avb.2013.07.003 PMC3763365

[13] Barros AJD, Menezes AMB, Santos IS, Assunção MCF, Gigante DP, et al (2008) O mestrado do Programa de Pós-graduação em Epidemiologia da UFPel baseado em consórcio de pesquisa: uma experiência inovadora. Revista Brasileira de Epidemiologia 11(1): 133–44.

[14] Instituto Brasileiro de Geografia e Estatística. Censo IBGE 2010. [cited 2012 November 20]; Available from: http://www.censo2010.ibge.gov.br/sinopse/index.php?uf=43&dados=1.

[15] Craig CL, Marshall AL, Sjostrom M, Bauman AE, Booth ML, et al (2003) International Physical Activity Questionnaire: 12-Country Reliability and Validity. Med Sci Sports Exerc 35(8): 1381–95. 12900694 10.1249/01.MSS.0000078924.61453.FB

[16] Haskell WL, Lee IM, Pate RR, Powell KE, Blair SN, et al (2007) Physical activity and public health: updated recommendation for adults from the American College of Sports Medicine and the American Heart Association. Med Sci Sports Exerc 39(8): 1423–34. 17762377 10.1249/mss.0b013e3180616b27

[17] Malavasi LM, Duarte MFS, Both J, Reis RS (2007) Escala de Mobilidade Ativa no Ambiente Comunitário - News Brasil: retradução e reprodutibilidade. Rev Bras Cineantropom Desempenho Hum 9(4): 339–50.

[18] Ewart CK, Suchday S (2002) Discovering how urban poverty and violence affect health: development and validation of a Neighborhood Stress Index. Health Psychol 21(3): 254–62. 12027031 10.1037//0278-6133.21.3.254

[19] Amorim TC, Azevedo MR, Halal PC (2010) Physical activity levels according to physical and social environmental factors in a sample of adults living in South Brazil. J Phys Act Health 7(2): 204–12. 10.1123/jpah.7.s2.s204 20702908

[20] Silva IC, Azevedo MR, Goncalves H (2013) Leisure-time Physical Activity and Social Support among Brazilian Adults. J Phys Act Health 10(6): 871–9. 23072766 10.1123/jpah.10.6.871

[21] Kesteren JV. Some main results on international comparison and trends: Results from the Internationa Crime Victims Survey and the European Survey on Crime and Safety. 2008 [12/11/2012]; Available from: http://ssrn.com/abstract=1117972.

[22] Brennan S. Canadians' perceptions of personal safety and crime, 2009. Otawa2011 [November 12, 2012]; Available from: http://www.statcan.gc.ca/pub/85-002-x/2011001/article/11577-eng.htm.

[23] Piro FN, Noss O, Claussen B (2006) Physical activity among elderly people in a city population: the influence of neighbourhood level violence and self perceived safety. J Epidemiol Community Health 60(7): 626–32. 16790836 10.1136/jech.2005.042697 PMC2566241

[24] Shenassa ED, Liebhaber A, Ezeamama A (2006) Perceived Safety of Area of Residence and Exercise: A Pan-European Study. Am J Epidemiol. 2006 163(11): 1012–7. 10.1093/aje/kwj142 16571742

[25] Florindo AA, Salvador EP, Reis RS, Guimarães VV (2011) Percepção do ambiente e prática de atividade física em adultos residentes em região de baixo nível socioeconômico. Rev Saude Publica 45(2): 302–10. 21412570 10.1590/s0034-89102011000200009

[26] Parra DC, Hoehner CM, Hallal PC, Ribeiro IC, Reis R, et al (2011) Perceived environmental correlates of physical activity for leisure and transportation in Curitiba, Brazil. Prev Med 52(3–4): 234–8. 21195726 10.1016/j.ypmed.2010.12.008

[27] Hallal PC, Reis RS, Parra DC, Hoehner C, Brownson RC, et al (2010) Association between perceived environmental attributes and physical activity among adults in Recife, Brazil. J Phys Act Health 7(2): 213–22. 10.1123/jpah.7.s2.s213 20702909

